# The Government of the Royal College of Surgeons

**Published:** 1914-04-25

**Authors:** 


### The Hospital

The Workers' Newspaper of Administrative Medicine and Institutional Life, Administration, National Insurance and Health.

No. 1452, Vol. LVI. SATURDAY, APRIL 25, 1914. PRICE ONE PENNY.

### THE GOVERNMENT OF THE ROYAL COLLEGE OF SURGEONS.

The private Society of the members of the above College has lately taken the step of circularising effectively all medical men holding the diploma of M.R.C.S.Eng. as a principal qualification. This is good tactics on the Society's part, the interests of its members being, as is well known, to secure representation upon the governing body, or Council, of the College. It is surprising how little the situation has altered in this respect in the last eight years, a period which has been so full of generic changes in the governance of other departments of human activity. Let us say at once that we have not necessarily sympathy with any and every movement, loosely called "democratic," which has been unreflectingly launched merely out of obedience to the prevailing trend of things. Each single movement has, in fact, to be judged upon its individual merits, and the present seems a good opportunity for considering those the Society of Members of the Royal College of Surgeons claims. In 1907 the annual report of the governing body now under criticism contained observations upon a copy of a memorial submitted by the Members' Society to the Prime Minister, and forwarded to the Council of the Royal College of Surgeons by direction of a high Governmental official. This memorial claims that by the original charter, which still holds good, the whole property of the College was vested in the members, the Fellows (as such) having no legal interest in it. The College was, therefore, managed by the Council on behalf of the members, but the latter had no vote in the election of that Council. The members did not, however, demand such a representation as they would be entitled to by their mere numbers—they recognised the position of the Fellows created by the charter of 1843—but proposed to increase the number of the Council to thirty-two, three-fourths of these to be elected as hitherto by the Fellows, but the remaining eight to be members, and elected by members. The replies of the Council to the suggestions thus authoritatively brought to their notice were that the College was a body corporate with perpetual succession, the property being vested in this body corporate; that the functions of the Council were academic and scientific, having no reference to the benefit of members of the body corporate, individually or collectively, and not being concerned with social and economic matters; that if representation were granted the large numbers of the members would be made a reason for agitation from time to time for fuller representation still. These replies will strike many as being a trifle reactionary, and certainly nothing has happened in the last seven years to make them sound less so. On the contrary, in fact. The sociological side of medicine has become immensely prominent. Simultaneously the actual disunion of the medical profession has been revealed. Academic bodies far older than the Royal College of Surgeons have been all the time extending wider and wider recognition to practical, modern needs. To come to more domestic matters, the rise of provincial universities has impoverished the schools of medicine at Metropolitan hospitals. The member of the Royal College of Surgeons is now virtually a pass man in surgery of the London University, but yet receives in return nothing in the way of graduate rank. It is a most widespread delusion among responsible laymen that the London medical diplomate is an inferior product, particularly on the theoretical, scientific side, unaware as they are that the library of the Royal College of Surgeons is worth all the other medical libraries in the United Kingdom put together, and more also. The ordinary Fellow, it may be granted, is better qualified to govern than the ordinary member. But all such propositions suppose antinomies. Mr. W. B. Yeats has well said, à propos of the claims of modern special knowledge, that "it is almost certain a man cannot be a successful doctor, or engineer, or Cabinet Minister, and have a culture good enough to escape the mockery of the ragged art student who comes of an evening sometimes to borrow a half-sovereign." The existing Council manages—*pace* the anatomists—its technical examinations admirably; but, as regards wider issues, it may well be supposed that in a larger number of counsellors there would be more wisdom. If a suggestion may be made, the leaders of the members' movement might be advised to appeal for help to prominent medical men who have made their way solely on the membership diploma. The late Mr. Buckston Browne was an instance in point, and, to mention only one living example, Sir Ronald Ross, whose disposition is well known to be anything but reactionary, might, if he chose, powerfully influence both professional and lay opinion. On the other hand, those who have, with a sturdy unselfishness, already done so much and so well, would also find it helpful to keep themselves well clear of sciolists whose foggy expressions of opinion can only darken counsel and cause adverse prejudice.

